# Consumer Perception of Inpatient Medical Services

**DOI:** 10.1371/journal.pone.0166117

**Published:** 2016-11-10

**Authors:** Satoko Izugami, Kozo Takase

**Affiliations:** Department of Research Development, Division of Public Health, Graduate School of Medical and Dental Sciences, Tokyo Medical and Dental University, Tokyo, Japan; Universite de Bretagne Occidentale, FRANCE

## Abstract

Although it is currently popular to reflect consumers’ perspectives to medical service management, insufficient attempts have been made to understand detailed perception of the consumer side of medical services to promote medical services’ evaluation from the consumer viewpoint. The aim of this study was to descriptively reveal how consumers perceive medical services that they receive, focusing on inpatient medical services. We conducted semi-structured interviews with 10 adults who experienced hospitalization of five or more days. Constant comparative analysis was performed on the obtained descriptive data. We identified 1) medical procedures, 2) explanations from medical professionals, 3) behavior of medical service providers, 4) somatic sensations, and 5) self-perceived physical conditions as target factors that medical service consumers perceived during hospitalization. The response to the perceived target factors, “compared with the expectation that the consumer had before the hospitalization,” suggests that it is an important medical service consumer reaction to check if the service met their expectations for perceived factors. The response to the medical services perception targets suggested that medical service consumers are involved in medical services and interested in various perception targets. The expectations that medical service consumers have prior to hospitalization can largely influence inpatient medical services evaluation.

## Introduction

The importance of consumers’ perspectives is popular in medical service management. As noted in service management and service marketing areas [1–5], in service production, service consumer participation is essential. For medical services, patients are the consumers. The decision-making process regarding the medical services to be received by the customer usually contains the following components: judgments of health conditions, collection of information regarding optional medical service contents for the customer, and selection of the most optimal medical service for the customer. The judgment and selection involved in this process requires highly specialized medical knowledge. For general service areas that do not require highly specialized knowledge, customers play a role freely deciding as to whether service content meets their purchase motivation and objectives. On the other hand, in professional services such as medical services that require specialized knowledge, professionals are the service providers involved in the identification of consumers’ service needs, facilitating the consumers’ participation in the service production activities.

Although there are numerous research reports evaluating medical services, survey results for patient satisfaction, commonly used as a clinical assessment of consumers’ perspectives, have not contributed much to specific solutions leading to medical service improvement. Patient satisfaction has been globally used as one of the clinical indicators of structure, process and outcome, which is the framework proposed by Donabedian [6–9]. In patient satisfaction assessment, questions are generated from the medical service providers’ perspective and medical service consumers answer the questions. The limitation of the patient satisfaction survey has been pointed out to utilize as evidence for a need to improve service quality [10–12], because the measurement of patient satisfaction captures only several aspects of patients’ multifaceted experiences [11]. Since the Institute of Medicine in the USA stated patient-centered care in the area of healthcare [6], the focus and goal of medical service management has shifted from quality of care only, to both patient–centeredness and quality of care.

Consumers’ perceptions of received medical services may help service providers learn about consumers’ experiences of their services. The importance of service assessment from consumers’ perspectives has been noted in the business administration research area. Parasuraman and colleagues developed an assessment tool for service quality, SERVQUAL [13], to help understand the gap between consumers’ service expectations and of actual service quality [5, 13]. Recently, in addition to research reports of medical service assessments using SERVQUAL, an increasing number of studies have focused on patient perceptions and experiences as consumer’s perspectives [8, 14–26]. For example, in the Hospital Consumer Assessment of Healthcare Providers and Systems (HCAHPS) Hospital Survey conducted in the USA, consumers answer the questionnaire that measures medical service experiences based on patient perceptions soon after they experience medical services [18–20]. The survey compared the results among participating institutions since 2008 [22–25]. The questionnaire survey focused on patients’ experiences and satisfaction, and has been used in the other countries to evaluate medical services in hospitals [27]. Patients’ perceptions regarding experienced medical services can provide useful suggestions, not only for delivering patient-centered care, but also for improving the quality of care.

Although questionnaire-based surveys have many benefits, detailed consumers’ perceptions of specialized medical services are poorly understood because it is difficult to collect information that has not been included in the prepared questionnaire. To specifically determine consumers’ perceptions of medical service, it is necessary for consumers to express perception freely from their perspectives. Therefore, this study’s objective was to investigate consumers’ perceptions of medical service. Although there are various categories of medical service, we focused on inpatient medicine where medical service consumers stay longer in the service.

## Materials and Methods

In order to investigate specific consumer perceptions of inpatient medical services, we selected a descriptive qualitative research design based on personal interviews using a semi-structured interview guide (see S1 Appendix). Qualitative research is employed to understand various social processes and complex phenomena such as daily-life experiences and human interaction in healthcare settings [28, 29]. To explore consumers’ perceptions of inpatient medical services with precision, in-depth personal interviews were used. We conducted semi-structured interviews to capture vivid descriptions by our research participants, voicing their experiences of being inpatients.

### Sampling

Participants were adults who were able to complete a 60-min personal interview in Japanese, experiencing hospitalizations of five days or more. We excluded patients in obstetrics, cosmetic surgery, psychiatry, and emergency hospitalization.

Participant recruitment was conducted at the study sites (two general hospitals located in an urban area of Japan). The researchers (the interviewers in the personal interview) obtained informed consent, explaining the study nature to patients who showed interest in participation, using an explanatory form. The choice of study participation was re-confirmed prior to the interview on the interview date. The patients were regarded as participants when they completed the interview after giving written consent for study participation.

### Procedure

Personal interviews were scheduled at the participant’s convenience and conducted in a private room where privacy was assured. After obtaining participant age, gender, and number of hospitalizations, a semi-structured interview regarding the medical service they received during the latest hospitalization was conducted for about 60 min. During the interview, the interviewer encouraged participants to express anything that they had experienced during the hospitalization. Interviewers took note of observed gestures, body parts to which the participant pointed, and their expressions during the interview. The interview was voice recorded with participant consent and the interviewers generated a verbatim record, based on the voice recording. The notes on the participants’ observed gestures and expressions were added to the verbatim record. Specific names of persons and institutions within the voice record were recorded as alphabet letters or general nouns. The interviewer reviewed the complete verbatim record for the accuracy of the descriptions, by reading the verbatim record while listening to the voice record. The document file of the verbatim record was coded with eight number digits, indicating interview dates.

### Ethical Considerations

This study was conducted according to the principles expressed in the Declaration of Helsinki. The Ethical Committee at Tokyo Medical and Dental University, Department of Medicine, approved the present study (Approval Number 1875). Participants were recruited after the approvals of the Institutional Review Board were obtained at each study site; Approval Number 925 and Approval Number 14-R169.

Explanations were provided both verbally and in writing, regarding participants’ choices taking precedence, the agreement regarding their ability to withdraw after agreeing to participate in the study, not being disadvantaged by withdrawal from the research, and the way to withdraw from the research. Information regarding hospitalization services was collected only based on the participant interview and the researchers did not access the medical records from the study sites in general hospitals.

### Data Analysis

Data analysis was conducted concurrently with data collection, based on the constant comparative method, as recommended by Strauss and Corbin [30]. The generated verbatim record was analyzed for each participant in the order of the interviews.

As the verbatim record for each participant was thoroughly read, phrases in which the participants talked about inpatient medical services were highlighted by underlining, and the data were sectioned according to content. When the sectioned data included a demonstrative pronoun, the data section was labeled with a data number, adding demonstrated contents with round brackets after the demonstrative pronoun. The data number was generated by combining an alphabet letter (A–J) that indicated participants and the sequence (number) of the comments in the verbatim record, starting from 1 (e.g., A1).

During reading, a data section with the data number, a data label (a word frankly expressing the content of the description) was allocated for the section. By alternately reading the data section and data label, paying attention to when (i.e., before, during or after) the perception of the inpatient medical service occurred, we extracted data sections and labels that included contents perceived during the hospitalization. We repeatedly compared data sections, paying attention to words that described perception target input, categorized similar items into a group, and named the categories. Verbatim records for following cases were also analyzed with the same procedures and compared with the previously analyzed cases. Data collection and analysis were conducted under the supervision of one nursing researcher and one sociologist, both of whom specialized in qualitative research.

### Participants

Ten adults (six men, four women) who experienced hospitalizations of five days or more participated in this study. The age range of the participants was 28–84 years old (average: 63.5 years old). The range of number of hospitalizations was 1–10 (average 4) and it was the first admission to the study site for three participants. The interview was conducted once for each participant, between February and October 2015. The day of the interview ranged from one day before to 47 days after discharge; the total interview duration time ranged from 38–93 min (average 64.5 min).

## Findings

The experiences of inpatient medical services, obtained from 10 adults who experienced inpatient medical services, were categorized according to the perception target during hospitalization input and response to the perception target output (Table 1).

**Table 1:** Perception target of inpatient medical service during hospitalization and response after the perception.

Perception target		Response to the perception target<Output category>
{Input category}	“Input subcategory”	
Medical procedures	Consultation (B C E F G I J)	
	Surgery (B C D F G H I J)	Feel unpleasant (F)
		Have irresistible feelings (F)
		Compare expectations before the hospitalization (C F G H)
		Compare previous treatment experiences (C)
		Feels like a first lifetime event (F G H)
	Endoscopy (A E)	Have irresistible feelings (A)
		Compare expectations before the hospitalization (A E)
		Compare previous treatment experiences (A)
		Feels like a first lifetime event (E)
		Search for reasons (E)
	Anesthesia (B F G H J)	Compare previous treatment experiences (J)
	Examination (E)	
	Nursing (B H I J)	
	Medication (D)	Feels like a first lifetime event (I)
		Compare expectations before the hospitalization (I)
		Ask questions (I)
	Rehabilitation (B D I)	
Explanations from medical professionals	Explanation of the treatment progress and results (A B C E F H I)	Try not to think about it (C)
		Feel unconvinced (E H)
		Search for reasons (E)
		Compare expectations before the hospitalization (B C E G H)
	Explanation of required behaviors for the treatment (C G H I J)	Follow the instructions (C H I J)
		Compare expectations before the hospitalization (G I)
		Compare previous treatment experiences (C)
		Ask questions (I)
	Explanation of nurse call (A D E F G H)	Press the nurse call (C D E H I J)
		Do not press the nurse call (D E G)
Behavior of medical service providers	Behavior of medical doctors (B D E G I)	Observe (B D E G I)
		Search for reasons (B E G)
		Think that it cannot be helped (D E H)
	Behavior of nurses (A B D E G H I J)	Observe (A B D E G H I J)
		Search for reasons (A D E G H I)
		Think that it cannot be helped (A D E H)
	Behavior of nurse assistants (E)	Observe (E)
		Search for reasons (E)
		Think that it cannot be helped (E)
Somatic sensations	Pain (B C E F G H J)	Search for reasons (C E J G)
		Compare expectations before the hospitalization (E J)
		Compare previous treatment experiences (C)
		Think that it cannot be helped (E G)
	Nausea (B E)	Search for reasons (B E)
		Compare expectations before the hospitalization (E)
		Ask questions (E)
	Trembling of the body (H)	
	Malaise (C)	Compare previous treatment experiences (C)
	Sense of thirst (C J)	Compare previous treatment experiences (C)
	Sense of being connected to medical devices (G H)	
Self-perceived physical condition	Operative wound (I, J)	Compare expectations before the hospitalization (I)
		Seek the reasons (I)
	Bleeding (J)	Compare expectations before the hospitalization (J)
		Ask questions (J)
	Contents of tube connected to the body (F G H)	Have unpleasant feelings (F)
		Ask questions (G)
	Body being connected to medical devices (F G H J)	Feel unpleasant (F)

Alphabet letters within brackets in the table (A–J) indicates participant ID.

The target perception input, which was the consumer’s experience of inpatient medical services, was summarized into five categories. Findings are described in the following section in sequence of the input category indicated in Table 1. Input category is shown with {}, “” for input subcategory, and < > for output category. Interview data obtained from the participants were shown in italic font and the data number is indicated in []. Round brackets () within the interview data indicate supplement provided by the researcher.

### Category 1: Medical procedures

This is a group category of {medical procedures} that medical professions performed at the study site. This category included “Consultation,” “Surgery,” “Endoscopy,” “Anesthesia,” “Examination,” “Nursing,” “Medication,” and “Rehabilitation.”

For medical service consumers, “surgery” and “endoscopy” were events that led to <having irresistible feelings> and <feeling unpleasant>.

Regarding the surgery, after all, it is like being a fish on the cutting board… [A8]

I really did not want (to undergo the surgery)… Although it was exactly as it was explained, I really did not want to…But it was easier than I thought. There is not much I could do, because I was like a fish on a cutting board. It was not something I could resist. I felt “do whatever you want” after all. [F10, 16]

The {medical procedures} that the medical service consumer received for the first time are <felt like a first lifetime event> and <compared with expectations before the hospitalization>.

I think total duration of the surgery was about the same as it was planned and explained; the surgery was 4 to 5 hours long with an extra 2 hours for the preparation and cleaning up afterwards. [G7]

(In this hospitalization), the doctor told me that the tumor should be removed as soon as possible and it became a large-scale thing. I think that to undergo surgery might always be like this. I think this is normal. This is the first surgery for me and I cannot really judge what is good and bad [H59, 69]

For the medical service consumers who received similar {Medical procedures}, {Medical procedures} they received this time were <compared with previous treatment experiences> and <compared with expectations before the hospitalization>.

When the doctor explained (the treatment) to me, thinking about the previous treatment, I can imagine what will be painful… Now I know the procedure of the surgery. After this, they will do this, this, and so on. When I remembered that this is my fifth hospitalization, I thought that I have never worried about the surgery may not work. [C50, 86,111]

The consumers who received {Medical procedures} <compared with expectations before the hospitalization> and when they encountered unexpected situations, they <searched for reasons>.

#### First patient who encountered an unexpected situation

The doctor who performed the endoscopy was different from the one I expected. I thought “What?” I thought the doctor who explained the treatment plan was the one who performed the surgery…I did not know that until I lay down and (the endoscopy) was started. I didn’t know that. The doctor who I expected to perform the surgery kept standing beside and supervising the doctor performing the surgery. The doctor kept on instructing (things) to do this and that. Is this because the surgery was not very complicated? Or is it for experience for the young doctor? I believe they should not do that (to let the young doctor perform the surgery) if the surgery is difficult. Probably because this was an easy case, the young (doctor) was performing the surgery, for a kind of practice. If it is the case, I thought that’s fine…I mean, probably it (myself) is not that difficult a case. [E55, 56, 65]

#### Second patient who encountered an unexpected situation

Intravenous drip was continued three days after the surgery and oral medication started afterwards instead. The daily dose of the medication was kind of maximum. I mean, a very strong dose was prescribed. I had severe constipation due to the oral medication. I had never experienced constipation before. My stomach felt heavy at four days after the surgery…I felt very nauseous and could not do anything. I did not imagine suffering that much. It was very, very hard. It was so hard that it might have been harder than the surgery. [I21, 43]

### Category 2: Explanation from medical professionals

Category {Explanation from medical professionals}, conducted for medical service consumers during their hospitalization, comprised “explanation of the treatment progress and results,” “explanation of required behaviors for the treatment,” and “explanation of nurse call.”

Medical consumers who received “explanation of the treatment progress and results” <compared with expectations before the hospitalization>.

I really think that it (undergoing the surgery) was the right decision. I should have done it earlier. (Before the hospitalization) I had a very bad impression about artificial hip joints. I was scared when I imagined how it was going to be after implanting an artificial hip joint…[B11,12]

The biggest concern about the surgery complications were incontinence and urine leakage. I was relieved after the explanation that, although there is an individual difference, those symptoms gradually improve. I had mild symptoms the first day after the surgery, while after the second day, I had no problem at all. I became a little optimistic about enjoying the rest of my life after I was told (by the doctor) that the hospitalization treatment was finished as it was planned. [G29, 30, 81]

As a result of “explanations of treatment progress and results,” medical consumers <tried not to think about it>.

Well, I don’t think about it too much…you know. Rather than being worried about many things in advance, I want to cherish the feeling of fun…not exactly fun but beauty and joy…I try not to think about it too much. Rather than thinking about it (expected survival rate explained by the doctor) too much in advance, after all, I found being joyful is the most important. [C124, 131]

For medical service consumers, when “explanation of treatment progress and results” were made when they encountered an unexpected situation, they <feel unconvinced> with the explanation. Furthermore, although they <search for reasons> for the explanation, they sometimes <felt unconvinced> to a greater extent.

#### Third patient who encountered an unexpected situation

Although I consulted my doctor about the pulling sensation (at the operative site), he said to me “I have never heard of patients who still have the sensation one week after the operation” and “you may be too sensitive.” The doctor even told me three times during the hospitalization, “you may be more sensitive about pain than other patients.” Hmmm…”Sensitive?” I felt suspicious when my doctor told me “you feel the pain because you are too sensitive” when my pain was at its peak. The doctor may have never seen such a patient (who had abdominal pains and fever after ESD (Endoscopic Submucosal Dissection); while I have not confirmed it (with the doctor) yet, I think he had never seen a case of patient like me who had fever. This is because he only told me about the abdominal pains, saying, “In general, people do not have abdominal pains.” [E36–38]

Medical consumers who received “explanations of required behaviors for the treatment” <followed the instructions>.

After I walked to the restroom, they told me to walk around outside my hospital room, although it was painful. They told me that I must walk even though it is painful. After they removed the urinary tube, I walked around since they told me “It is good for your recovery.” [H39, 40]

Medical service consumers who had ever experienced similar {Medical procedures} <compared previous treatment experiences> with <explanations of required behaviors for treatment >.

I was not allowed to drink or eat after 9 PM the day before (the surgery). But the time (for the surgery) was changed; since this surgery was scheduled in the afternoon, I was allowed to drink some water and tea until noon on the day (of the surgery). I felt much easier than before. [C73]

Medical service consumers who received “explanations of required behaviors for treatment” prior to the surgery <followed the instructions> and <compared expectations before the hospitalization>. Consumers who encountered an unexpected situation <asked questions>.

#### Fourth patient who encountered an unexpected situation

I took medication for my chronic disease every Saturday; I was told, “Do not take the medication a week before the hospitalization.” I was told to bring all regular medication when I was hospitalized. Although I expected that I would take the medication for my chronic disease after the surgery, I was not allowed to do so. On Saturday during the hospitalization, I asked one nurse, “Why don’t you give me the medication?” and the nurse answered, “I will ask the doctor,” but the answer was very late. Then, I asked several times; after a few days, I was told (by the doctor) “You cannot take the medication during the hospitalization.” [I27, 29, 31, 32]

There were two different reactions of medical service consumers who received “explanations of nurse calls” to <press the nurse call > and <not press the nurse call>. Three cases of patients who <did not press the nurse call > are shown below.

#### First patient who <did not press the nurse call>

Nurses told me “Please call us anytime for anything in need” and “Please do not hesitate to press the call button and call us.”…However, when they (nurses) were busy, they could not come immediately. I had to wait for a long time when I wanted to go to the washroom during the night…I thought I might have an accident. I went to the washroom by myself without calling them (nurses). [D38, 39, 43]

#### Second patient who <did not press the nurse call>

About two days after the surgery, my abdomen was so painful that I was wondering what the cause was. I could not call nurses to ask, “What is this pain?” or “It is very painful.” Should I call nurses saying “It is painful”? They told me “Call us anytime you want” every time they left the room; even though they told me so, it’s a little, a little awkward to do so. The nurse call is a little awkward to press. Of course I could call them, but because they (nurses) come and visit our room often, I thought I could ask them when they come. [E87, 94, 95]

#### Third patient who <did not press the nurse call>

I didn’t press the nurse call. In my case, I asked all questions I wanted to ask when nurses were around, like “How about this?” In that way, they (nurses) told me “That’s alright” and “It depends, but it is because of this and that.” [G61]

### Category 3: Behavior of medical service providers

{Behavior of medical service providers} that medical service consumers perceived is a category comprising “behavior of medical doctors,” “behavior of nurses,” and “Behavior of nurse assistants.”

Medical service consumers <observed> encountered {behavior of medical service providers}. They <searched for reasons> for the observed {behavior of medical service providers}.

Nurses are too busy, as far as I observed. There was no one (nurse) when the nurse calls are ringing. Probably, that is the same for all hospitals. After all, it may be partially because patients are pressing the nurse call as they want (laugh)… [A23, 24]

It sometimes took some time for them (nurses) to come. Well, I understand that they are busy with other things. [D13]

When I was in the hospital, I was really amazed to see how doctors and nurses are working in practice. They looked busy. I thought it is a very hard working profession in general. They have many patients, too. Doctors have to perform surgeries, not only consultations; they have to stay at hospital. They have to consult on various patients even the day after outpatient surgery. It is a very physically demanding profession. [G41–43]

Although I understand that they (nurses) are busy, they are not gentle; they quickly finish their job since they are busy and leave immediately. During holidays, probably because they take shifts, there were fewer nurses. They are especially busy during such days. Usually, the staff is full, especially during the day. [H74, 75]

Although medical service consumers <search for reasons> for {behavior of medical service providers} that they <observe> during their hospitalization, they sometimes <think that it cannot be helped>.

Doctors and nurses may all be too busy. They occasionally enter the room and before we think, they leave our room and enter next room as soon as they finish their duty. I feel a little frustrated with it. I wish if they could give us a bit more time. Although nurses are kind, they are busy (talking with sigh). [D55, 57]

I think it cannot be helped that the number of nurses are insufficient; there are nurses who don’t understand my condition well. I have really looked up to nurses since I was hospitalized. I respect people who wish to be nurses. It (nursing) is a really hard job and I feel respect for them.[E84, 110]

When my doctor visited me, he always opened the curtain next to the bed a little and looked at me from between the curtain, saying “How are you?” The doctor then closed the curtain and left. He rarely entered inside the curtain. Is it awkward to enter? I had a good impression of the doctor, being kind of young and fresh. “How are you?” “Oh I see” (pantomimes the scene where the doctor closes the curtain and leaves quickly). This may be a bit exaggerated, but it was roughly like this (laugh). I should have been more prepared, but I am slow and the doctor moves so quickly. So I was like “Oh…he’s gone”… [E112, 114,115]

I wonder who the person was. I was once left alone (laugh). I think I was not told anything (by the person). I though the person was not qualified for anything. I think the person was assisting nurses, but it was awful. But I thought it could not be helped since the person was just an assistant, not a nurse. [E130, 132]

### Category 4: Somatic sensations

The category of the medical service consumer’s own {somatic sensations} during the hospitalization was summarized. It comprised “pain,” “nausea,” “trembling of the body,” “malaise,” “sense of thirst,” and “sense of being connected to medical devices.”

Medical service consumers who felt “pain” <searched for reasons> and “compared expectations before the hospitalization.”

Although I had almost no pain related to the surgery, my back was very painful because of immobilization. I was not allowed to roll on the bed. I didn’t complain about it (pain in back). This is because I had an explanation for it and I expected it. I knew that I had to bear it only for one night. [G9, 15]

Medical service consumers who had previous experiences of {Medical procedures} <compared previous treatment experiences> with present {somatic sensations}. They <searched for reasons> and <compared expectations before the hospitalization>.

Based on the previous surgery I had, on the first day after the surgery, my back was painful due to immobilization, and on the second day, I started to have a fever. After the last surgery, I had fever of 38.2°C at its highest. The fever lasts about a week and gradually improves and the temperature becomes stable. That’s the pattern. But my doctor encouraged me “Don’t worry. More and more cancer cells are killed by the fever.” I was thinking that the more fever I have, the more I recover and the more cancer cells are killed. Having the fever is normal. Because I had a better physical condition, that was too easy. I started to have headaches as I kept talking (in previous treatment). This time I feel I am getting much better. [C75–80]

Medical service consumers who <searched for reasons> for “pain” not only <compared expectations before the hospitalization>, but also <asked questions> to confirm if they were in an unexpected situation. Medical service consumers who encountered unexpected “pain” <think that it cannot be helped>.

#### Fifth patient who encountered an unexpected situation

I had a sort of muscle soreness on the right side of my abdomen. My back and side of abdomen was painful…and a lot of things happened all together. Fever, pain in the side of my abdomen, and menstrual pain…and I was told “you may have bacterial infection” for bowel pain. I experienced a lot of pain at once. Anyway, from my side abdomen to this area (point with right finger through the navel to left side abdomen) was painful…It was very hard. I had no idea what was going on. I had no explanation for the fever and risk of infection in advance…But I also thought it could not be helped. [E31, 33, 34, 40]

#### Sixth patient who encountered an unexpected situation

Although the surgical wound was in my abdomen, I had severe pain here (points from left shoulder to right collarbone through sternum). I wondered why this area was so painful. As I felt the pain, I gradually started to feel tightness and difficulty in breathing, like a dull pain when I breathed deeply. I wondered why. I had pain from the chest to shoulder. I consulted with my doctor a day before discharge, saying “I have some pain.” During the medical consultation the afternoon of the same day, I thought that the pain was similar to muscle soreness. So I said “I have very severe pain around here (from left shoulder to right collarbone, through sternum), just like muscle soreness.” Nothing about the pain was particularly written (on the explanation form). After all, it may not have been helped since the pain occurred in the area that was not related to the surgery. [J72–74, 80]

### Category 5: Self-perceived physical condition

The category {self-perceived physical condition} comprised “operative wound,” “bleeding,” “contents of the tube connected to the body,” and “body being connected to medical devices.”

Medical service consumers <felt unpleasant> to see their “body being connected to medical devices.” Consumers <asked questions > when they were worried about “contents of tubes connected to the body.”

For about a day afterward, there was a urinary tube. It (The tube) must be inserted. Until it (the tube) was removed, which happened probably next day of the surgery, I felt very uncomfortable. [F12]

When I saw red things coming out through the tube inserted in my body, I was very worried. But the good thing is that they answered my questions, saying “This is because of that.” We have a lot of concerns since we don’t know much about medicine. For example, when I asked a nurse, “Is this normal amount of urine?” the nurse answered, “Oh, that’s fine. The color is also normal” or “It is normal.” These single phrases relieved me of my concerns. [G46–48]

Medical service consumers who saw “operative wounds” and “bleeding” <compared expectations before the hospitalization> felt the possibility of unexpected situations happening and <searched for reasons> why their body was in the {self-perceived physical condition}.

#### Seventh patient who encountered an unexpected situation

In addition to the scar of the operative wound, there was a kind of sore or blister. My guess was…I think it is a scar from the equipment that stabilized my leg. I wanted a detailed explanation for why the blister was formed, but there was no explanation. The doctor told me that there is a blister formed. But there was no explanation for the reasons why it (the blister) formed. [I71–73]

#### Eighth patient who encountered an unexpected situation

Because I had a sense of bleeding after the surgery, I consulted (my doctor) about it. But I was told (by the doctor) “there is no problem” and the doctor decided to discharge me. I don’t think there was any explanation about the possibility of the bleeding. Since (it was written that) sanitary napkins were included in the explanation form explaining items that I needed to bring for hospitalization, I predicted that I may have some bleeding. [J69–70]

## Discussion

The present study revealed five perception targets for inpatient medical services that medical service consumers experienced. Almost all consumers who participated in this research experienced inescapable physical and emotional suffering associated with [medical procedure]; they often made efforts to overcome their suffering. Both [somatic sensations] and [self-perceived physical conditions] showed multifaceted themes that consumers perceived physical and emotional suffering from their experiences of medical services. Consumers of inpatient medical services described their discomfort and anxiety regarding the perceived “operative wound” or “contents of tubes connected to body,” while medical professionals might evaluate to track recovery. The qualitative study highlighted patients’ experiences, also reporting on the physical and emotional needs of patients as one of the identified themes from the interview data [27, 31]. The suffering experienced by consumers during hospitalization was inextricably intertwined with their physical and emotional needs.

Each example case described a response to the perception targets for inpatient medical service; thus, medical service consumers are involved in medical service areas with various interests. Medical service is an interpersonal service that is provided in person, mainly by medical professionals [2]. In interpersonal services, service consumers meet service providers and they also work together to produce an aimed service if necessary [2–4]. Among {explanations from medical professionals} that participants received, “explanations of required behaviors for treatment” and “explanations of nurse call” were conducted to provide the aimed medical service safely; the fact that medical consumers were following explained instructions illustrates joint service production. Consumers’ activities as co-producers for their medical services were revealed as descriptions emerged of how patients engaged with medical professionals regarding the delivery process of their healthcare services [31]. Consumers’ perceptions of their involvements in medical services as that of co-producers may support the acquisition of experiential knowledge by consumers and the facilitation of patient-centered care by medical professionals.

Our study results have several implications for clinical practice. The observed behavior of patients, <do not press the nurse call>, in response to “explanation of nurse calls” is a typical example of behavior where medical service consumers did not follow the explained instructions. In one of the response behaviors when the nurse call was not pressed for unidentified pain after wondering whether the medical service consumer should press it, the consumer was told, “Please press nurse call in case you need anything.” In this case, it was likely that the medical service consumer did not understand the specific situations as to when the consumer should press the nurse call during “explanations for nurse call.” Information provided to consumers by medical professionals occasionally causes unexpected consumer responses, despite the informant’s intentions. In the situation where medical service providers explain expected behavior to medical service consumers, such as “explanation of nurse call,” specific explanations that help accomplish expected behavior have to be provided. Research articles describing information needs from consumers’ narratives revealed what consumers were uninformed about and how they accessed the necessary information during their experienced medical services [27, 32–34]. The provision of useful and clear information with consumer-oriented explanations to medical service consumers could help expand consumer involvement in their medical services, and foster a better relationship between patients as consumers and medical professionals as providers in medical service co-production.

Our study findings suggested that among five categories of target perception during hospitalization, four categories, excluding {Behavior of medical service providers}, included <compare expectations before the hospitalization>. Inpatient medical service consumers started hospitalizations with an expectation of inpatient medical services they were going to receive. In general, service theory suggests that consumers have expectations for the service when they receive it [1, 35, 36]. As our study results suggested, expectations prior to the hospitalization are the case for inpatient medical service consumers. In order to clarify expectations of inpatient medical service consumers, medical service providers need to focus more on expectations medical service consumers have prior to the hospitalization.

As a result of focusing on participants who encountered unexpected situations, we observed the process of participants starting to perceive that the situations were different from their expectations in all eight categories shown in the findings through <comparing expectations before the hospitalization> with target perception inputs during hospitalization. The consequence of the reaction of the inpatient medical service consumers to <comparing expectations before the hospitalization> includes evaluation of medical service perceived from the consumers’ perspectives during the hospitalization. The evaluation was conducted during the hospitalization; it is an evaluation of the process within the framework of the medical service quality assessment from the consumer’s perspective. Furthermore, because the expected situations prior to the hospitalization reflect consumer expectations, to <compare expectations before the hospitalization> is equivalent to the measurement targets of SERVQUAL that measure service quality by identifying the gap between service consumers’ expectations and actual experiences[5, 13]. Therefore, expectations of inpatient medical service consumers, which are the situations that medical service consumers predicted for inpatient medical services prior to the hospitalization, can influence medical service evaluations. Researchers who explored patient perspectives of medical services reported components not to be measured by questionnaire such as the HCAHPS Hospital Survey, based on the results of their own qualitative studies; they also reported necessity for more research examining the determinants of patients’ experiences in a hospital setting [11, 37, 38]. Although the present study collected and analyzed data focusing on perception targets during hospitalization, future study needs to address expectations prior to hospitalization, which are equivalent to the expectations of inpatient medical service consumers.

## Limitations and Future Directions of the Study

The present study results were collected from ten adults who experienced inpatient medical services. Because the data was collected using a 60 min interview of their remembered experience, any experiences that they did not remember during the interview could not be included in the study data even if the medical service was actually provided.

We could not evaluate if the collected data matched the actual medical service contents. In order to identify perception of service consumers more accurately, it is desirable to collect information about the contents of the actual medical services and to evaluate the consistency of the information and the perception of the service consumers.

## Conclusions

Understanding consumers’ perspectives of medical services from their experience is essential in ensuring that their medical services needs are met, and that patient-centered care is delivered. This descriptive study showed the perceptions of consumers of inpatient medical services, specifically, how consumers relayed their experiences of hospitalization. We analyzed the descriptive data obtained through in-depth interviews of 10 adults who had experienced hospitalization over five or more days. As a result of our analysis, we identified 1) medical procedures, 2) explanations from medical professionals, 3) behavior of medical service providers, 4) somatic sensations, and 5) self-perceived physical conditions, as perception target factors. Consumers’ perspectives represented diverse themes of physical and emotional suffering from their experiences of inpatient medical services, and the suffering became entwined with their physical and emotional needs. The activities of medical consumers after being given instructions by medical professionals showed consumers’ participation in their receiving of medical services as co-producers. Furthermore, the expectations that medical service consumers have prior to the hospitalization can largely influence the inpatient medical services evaluation. Further studies are needed to explore the expectations that medical service consumers have prior to the hospitalization, and to examine how expectations regarding the medical service affect their evaluation of received care.

## Supporting Information

S1 Appendix Interview guide of “Consumer perception of inpatient medical services”. (DOCX)
