# Supplementary material for: Consumer Perception of Inpatient Medical Services
Source: PLoS One. 2016 Nov 10;11(11):e0166117. doi: 10.1371/journal.pone.0166117 (PMC5104407; doi:10.1371/journal.pone.0166117)
Supplement: S1 Appendix — (DOCX) [file pone.0166117.s001.docx]

S1 Appendix

Interview guide of “Consumer perception of inpatient medical services”

I. Demographics of the participants (all participants)

1．What is your age?

2．Was this the first hospitalization for you?

YES

NO → Please tell me your experience(s) of previous hospitalization (s) [Subject to question III]

　　　　　　　　(Ask the number of hospitalizations, including in other medical institutions)

II. About this hospitalization (all participants)

1. What were the dates of admission and discharge?

2. What was the objective of this hospitalization?

　(or) what was scheduled examination and /or treatment (surgery, chemotherapy)?

3. Looking back from admission to discharge during this hospitalization, how many points do you give for your satisfaction with 100 being maximum?

Please tell me the reasons for the points you have given.

4. Did you have an explanation for the examination and / or treatment (the answers in 2.) when you were hospitalized from doctors and / or nurses?

［If YES,］

4-1 Can you tell me what explanation was provided, as far as you remember?

4-2 What did you think after you received the examination and / or treatment in practice, compared to the predicted condition based on the explanation?

(or)　How was it compared to the expectation you had before the hospitalization?

5. Tell me impressive events you experienced or felt during the hospitalization.

6. How many points do you give for service quality of your doctors (check the number of doctors in charge) with 100 being maximum?

6-1 Please tell me the reasons for the points you have given.

7. Please tell me the service of doctors that you liked.

8. Please tell me the service of doctors that you did not like.

9. How many points do you give for service quality of nurses with 100 being maximum?

9-1 Please tell me the reasons for the points you have given.

10. Please tell me the service of nurses that you liked.

11. Please tell me the service of nurses that you did not like.

12. Please tell me the service of the other hospital staff that you liked.

13. Please tell me the service of the other hospital staff that you did not like.

III. Ask the following questions only when this hospitalization is the second or more in the life of the patient (i.e., only patients who answered “NO” in I.-2.).

1. Patients who have been hospitalized only in this hospital.

Please rank the satisfaction for this hospitalization, including your previous hospitalization experiences (please provide the reasons).

1. Patients who have been hospitalized in other hospitals

Please rank the satisfaction for this hospitalization, including previous hospitalization experiences at other hospitals or other wards (please provide the reasons).

IV. Opinion toward future hospitalization (all participants)

1. If you need to be hospitalized, do you want to be hospitalized at this hospital again? (Please provide the reasons)

2. If your family needs to be hospitalized, do you recommend this hospital? (Please provide the reasons)

3. If your friend needs to be hospitalized, do you recommend this hospital? (Please provide the reasons)
